# Sudden blindness due to isolated sphenoid sinus mucocele and retention cyst

**DOI:** 10.5935/1808-8694.20130021

**Published:** 2015-10-14

**Authors:** Guive Sharifi, Maryam Jalessi, Dariush Erfanian, Mohammad Farhadi

**Affiliations:** MD, Assistant Professor of Neurosurgery, Neurosurgery Department, Loghman Hakim Hospital, Shaheed Behesht University of Medical Sciences, Tehran, Iran (Assistant Professor of Neurosurgery); MD, Assistant Professor in ENT and Head and Neck surgery, ENT-Head and Neck Surgery Research Center and Department, Rasool Akram Hospital, Tehran University of Medical Sciences, Tehran, Iran (Assistant Professor in ENT and Head and Neck surgery); MD, ENT and Head and Neck surgen, ENT-Head and Neck Surgery Research Center and Department, Rasool Akram Hospital, Tehran University of Medical Sciences, Tehran, Iran (ENT and Head and Neck surgen); MD, Professor in ENT and Head and Neck surgery, ENT-Head and Neck Surgery Research Center and Department, Rasool Akram Hospital, Tehran University of Medical, Tehran, Iran (Professor in ENT and Head and Neck surgery)

**Keywords:** ischemic optical neuropathy, mucocele, sphenoid sinus

## INTRODUCTION

Sphenoid sinus mucocele (SSM) caused by obstruction of sinus ostium while sphenoid sinus retention cyst (SSRC) is due to obstruction of mucinous gland ostium which could enlarge to obstruct the sinus ostium and lead to acute sinusitis or mucocele^1^, ^2^, ^3^.

Incidence of isolated SSM is about 2% out of paranasal sinus (PNS) mucocele, but there is no specifically occurrence rate regarding SSRc^2^^,^^3^. Although optic neuropathy has been reported with SSM, no such a report exists for SSRC.

We herein describe one case of SSM and two cases of SSRC with sudden unilateral visual loss that recovered following endonasal endoscopic sinus surgery.

## CASE PRESENTATION

### Case 1

A 25-year-old man with left-sided vision loss (light perception (LP), afferent pupillary defect (APD)) occurred within 24 hours, was referred after 10 days of the attack.

**Figure 1:**
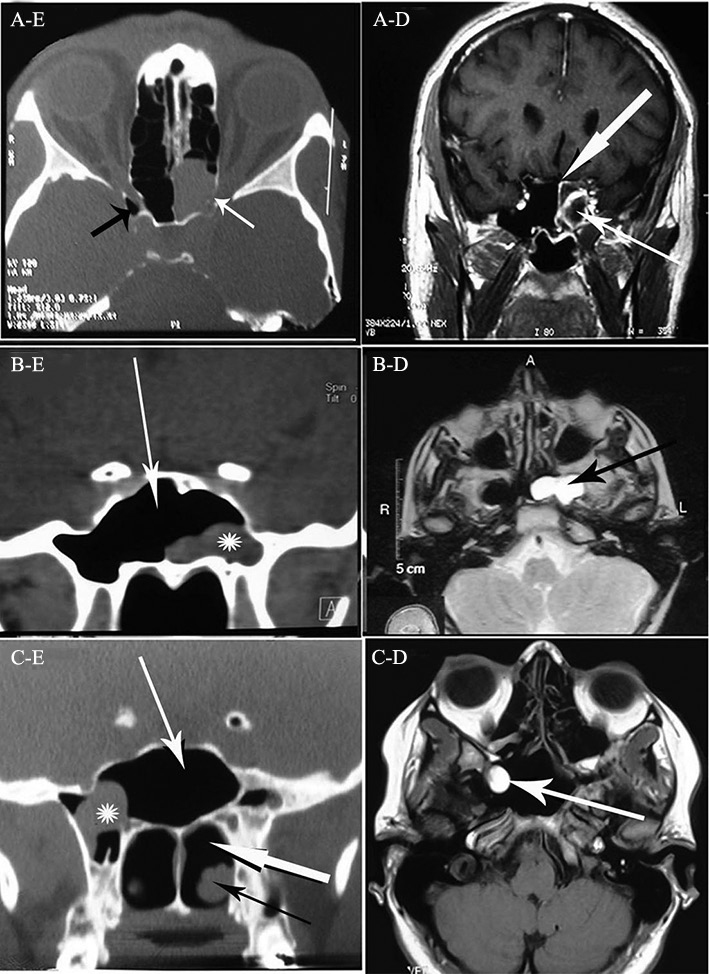
A: (Left) axial CT scan of sinuses shows complete opa-cification of the left sphenoid sinus with dehiscence in the left optic canal (white arrow), opticocarotid recess (black arrow); (Right) MRI shows the cyst with rim enhancement obstructing the sinus ostium (thin arrow), thick arrow shows enhanced sphenoid sinus mucosa. B: (Left) coronal CT scan of sinuses shows a retention cyst (astrix) in the lateral recess of sphenoid sinus, white arrow shows sphenoid sinus; (Right) T2-weighted MRI shows left sphenoid sinus hyper-intense retention cyst (black arrow). C: (Left) coronal CT scan shows sphenoid sinus (white thin arrow) and retention cyst (astrix) without pressured on the optic chiasm, posterior chornea (thick white arrow) and inferior turbinate (black arrow); (Right) MRI shows hyper-intense mass in the sphenoid sinus and its lateral recess (white arrow).

Magnetic resonance imaging (MRI) showed a mass with rim enhancement obstructing the sphenoid sinus ostium (Figure 1A). Computerized tomography (CT) dehiscence in the optic canal (confirmed intra operatively).

### Case 2

A 12-year-old boy with a history of gradual visual loss of left eye over 2 month-period was referred because of left sphenoid sinus mass in MRI with no Gadolinium uptake that was compatible with SSRC. There was no obvious compression of the optic nerve in the CTS or MRI (confirmed also during surgery).

### Case 3

(28-year-old woman) showed up with total visual loss (LP and APD) of the right eye over 34 day period and retro-orbital pain. She had a similar attack 4 years earlier treated with 2-week administration of Dexamethasone. MRI showed an RC in the sphenoid sinus (Figure 1B) touching the inferomedial border of the optic nerve.

All patients were referred by ophthalmologist with the first diagnosis of retrobulbar optic neuritis (RON) following incomplete recovery with administration of glucocorticoids. The patients underwent endoscopic endonasal approach and the cysts were removed. Total vision and visual field recovery were achieved in case 1 and 3 and partial recovery in second case.

## DISCUSSION

Isolated inflammatory diseases of SS are infrequent comparing to other paranasal sinuses. However neglected sphenoiditis could lead to critical complications by affecting adjacent vital structures such as cranial nerves II, III, IV, VI^2^, ^3^, ^4^, ^5^.

Visual loss and blindness often occur following circulatory disorders of the optic nerve caused by prolonged mucocele pressure that erode the bone and put the nerve in jeopardy^3^. Rapid spread of infection or inflammation can be another mechanism that seems responsible in our RC cases, as there was no pressure effect on the optic nerve or finding in favor of canal dehiscence.

Mucocele could show four patterns of MRI signal intensity according to the protein concentration revealing its chronicity^6^. The third case showed a hyperintense lesion in both T1W and T2W images that is compatible with greater than 30% protein concentration. This information together with a history of same attack 4 years earlier confirms the chronicity of the lesion. It has been stated that asymptomatic cases of RC in paranasal sinuses need no treatment except those with filling more than 50% of the sinus volume, fast growing mucoceles, and intracranial or mucocele involving the orbit^2^. According to our cases, it seems that there is a risk of optic injury in untreated cases of sphenoid RC. This urges us to consider surgery for RC of the sphenoid sinus opposing the other paranasal sinuses.

A high index of suspicion is needed for proper diagnosis of this entity especially for neurologists and ophthalmologists who are usually first physicians visiting sudden visual loss cases.
